# Prognostic Factors for Mortality in Acute Mesenteric Ischemia

**DOI:** 10.3390/jcm11133619

**Published:** 2022-06-23

**Authors:** Carlos Constantin Otto, Zoltan Czigany, Daniel Heise, Philipp Bruners, Drosos Kotelis, Sven Arke Lang, Tom Florian Ulmer, Ulf Peter Neumann, Christian Klink, Jan Bednarsch

**Affiliations:** 1 Department of Surgery and Transplantation, University Hospital RWTH Aachen, 52074 Aachen, Germany; caotto@ukaachen.de (C.C.O.); zczigany@ukaachen.de (Z.C.); dheise@ukaachen.de (D.H.); svlang@ukaachen.de (S.A.L.); fulmer@ukaachen.de (T.F.U.); ulf.neumann@mumc.nl (U.P.N.); chirurgie-sp@diakonissen.de (C.K.); 2 Department of Diagnostic and Interventional Radiology, University Hospital RWTH Aachen, 52074 Aachen, Germany; pbruners@ukaachen.de; 3 Department of Vascular Surgery, University Hospital RWTH Aachen, 52074 Aachen, Germany; dkotelis@ukaachen.de; 4 Department of Vascular Surgery, University Hospital Bern, 3010 Bern, Switzerland; 5 Department of Surgery, Maastricht University Medical Center (MUMC), 6229 HX Maastricht, The Netherlands; 6 Department of Surgery, Diakonissen-Stiftungs-Krankenhaus Speyer, 67346 Speyer, Germany

**Keywords:** acute mesenteric ischemia, lactate, morbidity, mortality

## Abstract

Postoperative mortality in patients undergoing surgical and/or interventional treatment for acute mesenteric ischemia (AMI) has remained an unsolved problem in recent decades. Here, we investigated clinical predictors of postoperative mortality in a large European cohort of patients undergoing treatment for AMI. In total, 179 patients who underwent surgical and/or interventional treatment for AMI between 2009 and 2021 at our institution were included in this analysis. Associations between postoperative mortality and various clinical variables were assessed using univariate and multivariable binary logistic regression analysis. Most of the patients were diagnosed with arterial ischemia (AI; *n* = 104), while venous ischemia (VI; *n* = 21) and non-occlusive mesenteric ischemia (NOMI; *n* = 54) were present in a subset of patients. Overall inhouse mortality was 55.9% (100/179). Multivariable analyses identified leukocytes (HR = 1.08; *p* = *0*.008), lactate (HR = 1.25; *p* = 0.01), bilirubin (HR = 2.05; *p* = 0.045), creatinine (HR = 1.48; *p* = 0.039), etiology (AI, VI or NOMI; *p* = 0.038) and portomesenteric vein gas (PMVG; HR = 23.02; *p* = 0.012) as independent predictors of postoperative mortality. In a subanalysis excluding patients with fatal prognosis at the first surgical exploration (*n* = 24), leukocytes (HR = 1.09; *p* = 0.004), lactate (HR = 1.27; *p* = 0.003), etiology (AI, VI or NOMI; *p* = 0.006), PMVG (HR = 17.02; *p* = 0.018) and intraoperative FFP transfusion (HR = 4.4; *p* = 0.025) were determined as independent predictors of postoperative mortality. Further, the risk of fatal outcome changed disproportionally with increased preoperative lactate values. The clinical outcome of patients with AMI was determined using a combination of pre- and intraoperative clinical and radiological characteristics. Serum lactate appears to be of major clinical importance as the risk of fatal outcome increases significantly with higher lactate values.

## 1. Introduction

Acute mesenteric ischemia (AMI) is a an often rapidly progressing clinical condition, which is commonly diagnosed late and associated with dismal outcome after surgical or endovascular therapy [1,2,3]. While a rapid diagnosis and subsequent treatment result in distinctively better outcomes [4,5], the overall mortality rates are high in comparison to other surgical emergencies [6]. Therefore, patients suspected of AMI should be diagnosed and treated with high priority to achieve acceptable outcomes [5,7]. Unfortunately, the lack of specific parameters and often vague early clinical symptoms frequently result in a notable delay in diagnostic measures and targeted treatment [8].

A variety of risk factors for adverse outcomes have been identified in the literature, with a prolonged duration of symptoms before specific treatment being the most prominent negative predictor, as described previously [9]. Additionally, individual patient characteristics as basic demographics and comorbidities as well as laboratory values have been under the spotlight of interest in recent decades [10,11,12,13]. Interestingly, the group of patients presenting with AMI is also heterogenous, as the underlying conditions and corresponding subtypes, e.g., arterial ischemia (AI), venous ischemia (VI) and non-occlusive mesenteric ischemia (NOMI), display different features and distinct outcomes [14].

Given the highly heterogenous nature of AMI and its relatively rare occurrence, only a limited number of monocentric series are available for investigating the disease. Moreover, those studies vary in design, endpoints, and findings [10,15,16], resulting in overall inconsistent and low-quality evidence. To further explore potential prognostic factors of surgical morbidity and mortality, we analyzed clinical outcomes in a European cohort of patients undergoing treatment for AMI.

## 2. Materials and Methods

### 2.1. Patients and Definitions

This study comprised one hundred seventy-nine (*n* = 179) consecutive patients diagnosed with AMI and treated with surgery between 2009 and 2021 at a large academic tertiary referral center (University Hospital RWTH Aachen (UH-RWTH)). Inclusion criteria were (a) patients undergoing surgical/endovascular treatment after the radiological diagnosis of AMI. Exclusion criteria were: (a) no present AMI during surgical exploration and (b) patients deceasing prior to surgical exploration and (c) patients refusing to undergo surgical/endovascular treatment. The study was further conducted in accordance with the requirements of the Institutional Review Board of the RWTH-Aachen University (EK 334/21), the current version of the Declaration of Helsinki, and the good clinical practice guidelines (ICH-GCP). In this retrospective study, AMI was defined as the occurrence of an abrupt cessation of the mesenteric blood flow leading to malperfusion of the bowel with associated and acute symptoms and eventually bowel necrosis [8]. AI was assumed in cases with arterial obstruction due to atherosclerotic disease, atherothrombosis, arterial dissection or arterial embolism, while VI was diagnosed in cases with thrombosis of the mesenterial veins. NOMI was present in patients with low blood states as a consequence of circulatory failure and no apparent vascular occlusion.

### 2.2. Standard Clinical Management of AMI Patients

AMI was diagnosed based on the clinical condition of the patient, blood values and cross-sectional imaging. After diagnosis, treatment was facilitated in terms of an interdisciplinary approach always involving a team of experienced visceral and vascular surgeons, as well as interventional radiologists. In cases of AI with acute arterial occlusions of the superior mesenteric artery (SMA) and/or the celiac trunk (TC), endovascular or open revascularization was carried out before or after subsequent bowel resection. The decision for endovascular or open revascularization and the therapeutic sequence was made on a case-by-case basis. In the endovascular approach, which was executed in the radiological department, the occluded vessel was recanalized via balloon angioplasty and balloon-expandable stenting (Formula^®^ 535 Vascular Balloon-Expandable Stent, Cook Medical; Omnilink Elite Vascular Balloon-Expandable Stent System, MULTI-LINK VISION RX Coronary Stent System, Herculink Elite^®^, Abbott Vascular, Chicago, IL, USA) via a femoral or brachial artery access. In the case of two occluded vessels (usually SMA and TC), the SMA was preferably recanalized and the TC only if SMA was not possible to be recanalized, as previously described [17]. Dual antiplatelet therapy with acetylsalicylic acid and clopidogrel was administered after recanalization. Open revascularization was usually carried out using conventional thrombectomy via a Fogarty catheter and following intraoperative local heparin application and/or—if thrombectomy was unsuccessful—through surgical bypass utilizing autologous vein or prosthetic grafts in an antegrade or retrograde fashion on a case-by-case basis (Table 1). In VI, the therapy of choice was immediate therapeutic anticoagulation and surgical exploration.

In cases of AMI due to NOMI or VI (except for one case with conventional thrombectomy), no revascularization was carried out. Operative exploration was performed in every patient. All abdominal organs were carefully examined regarding signs of ischemia and were (partially) resected if no recovery was expected. Primary fascial closure was always preferred if feasible; however, in cases with elevated abdominal pressure, temporary abdominal closure with a prosthetic mesh in inlay position was conducted. Further, second look exploration was carried out per the protocol in every patient after 24 h to ensure sufficient radicality and treatment success.

### 2.3. Data Extraction and Quality Management

All relevant patient data were extracted from the electronical case records including preoperative characteristics, operative procedures, and postoperative outcome. Every cross-sectional imaging was also re-analyzed for signs of portomesenteric vein gas (PMVG), pneumatosis intestinalis (PI), ascites, bowel distension, bowel wall thickening and pneumoperitoneum by an experienced staff radiologist.

### 2.4. Statistical Analysis

The primary endpoint of this study was in-hospital mortality in AMI patients undergoing treatment. Categorial data are shown in the form of numbers and percentages. Data derived from continuous variables are presented as the median and inter-quartile range. Associations between perioperative variables and the primary endpoint were assessed by means of binary logistic regressions. Variables showing a *p*-value < 0.05 in univariate analysis were subsequently transferred into a multivariable model and analyzed with multivariable binary logistic regressions using backward elimination. For this purpose, nominal and categorical data were recoded into a scaled dummy variable. The level of significance was set to *p* < 0.05, and *p*-values are given for two-sided testing. Analyses were performed using SPSS Statistics 24 (IBM Corp., Armonk, NY, USA).

## 3. Results

### 3.1. Preoperative, Operative and Postoperative Data

A total of 179 patients with a median age of 71 years (range: 61–80) and median body mass index (BMI) of 26 kg/m^2^ underwent surgery for AMI at our institution from 2009 to 2021. In the whole cohort, 104 patients (58.1%) presented with AI, 21 (11.7%) with VI and 54 (30.2%) with NOMI as underlying etiology. Of note, most patients (91.1%, 163/179) had a preoperative performance status of ASA III or higher, assessed by the attending anesthesiologist. While in most of the cohort (79.9%, 143/179), bowel resection was carried out, only 71 patients (39.6%) underwent open or endovascular revascularization. A total of 100 patients (55.9%) deceased during hospitalization, with 61 patients (58.7%) in the AI subgroup, 3 patients (14.3%) in the VI subgroup and 36 patients (66.7%) in the NOMI subgroup. Of note, a relevant subset of these patients (24/179, 13.4%) displayed a complete intestinal ischemia with a dismal prognosis during initial surgical exploration and were referred for palliative treatment. Almost all patients (177/179) showed postoperative complications, while a large proportion of the cohort (164/179; 91.6%) experienced major postoperative complications (Clavien Dindo ≥ 3). Further, a subanalysis comparing patients that had been revascularized to patients without revascularization showed no difference in major morbidity (Clavien Dindo ≥ 3, *p* = 0.343) or in-hospital mortality (*p* = 0.963, Supplementary Table S1). Detailed clinicopathological and perioperative characteristics are outlined in Table 1.

### 3.2. Univariate and Multivariable Analysis of Postoperative Mortality

A univariate binary logistic regression was carried out for postoperative mortality including all available pre- and intraoperative variables (Table 2). Here, age (HR = 1.02; *p* = 0.04), ASA (HR = 20.89; *p* = 0.004), leukocytes (HR = 1.04; *p* = 0.025), lactate (HR = 1.45; *p* < 0.001), hemoglobin (HR = 0.90; *p* = 0.048), bilirubin (HR = 1.60; *p* = 0.026), alkaline phosphatase (HR = 1.01; *p* = 0.034), prothrombine time (HR = 0.97; *p* < 0.001), INR (HR = 2.13; *p* = 0.012), etiology (*p* = 0.001), PI (HR = 2.74; *p* = 0.007), PMVG (HR = 18.25, *p* = 0.005), bowel distension (HR = 1.99, *p* = 0.03), extent of resection (*p* = 0.003) and FFP transfusion (HR = 3.75; *p* = 0.001) were associated with postoperative mortality (Table 2).

Variables showing a *p*-value < 0.05 in univariate analysis were further included in a multivariable binary logistic regression. In this multivariable model, leukocytes (HR = 1.08; *p* = 0.008), lactate (HR = 1.25; *p* = 0.01), bilirubin (HR = 2.05; *p* = 0.045), creatinine (HR = 1.48; *p* = 0.39), etiology (*p* = 0.038) and PMVG (HR = 23.02; *p* = 0.012) were determined as independent predictors of postoperative mortality.

To further explore the validity of predictors of postoperative mortality, a similar multivariable analysis regarding postoperative mortality was carried out excluding patients who presented with a dismal situation during initial surgical exploration and, therefore, referred to palliative care. In the corresponding multivariable model, leukocytes (HR = 1.09; *p* = 0.004), lactate (HR = 1.27; *p* = 0.003), etiology (*p* = 0.006), PMVG (HR = 17.02; *p* = 0.018) and intraoperative FFP transfusion (HR = 4.4; *p* = 0.025) showed independent significance (Table 3).

As lactate showed significance in both multivariable models, we further analyzed its prognostic role in univariate analysis, dividing the cohort into subgroups according to preoperative lactate. Here, the preoperative lactate value was strongly associated with the likelihood of fatal outcome in our cohort (Table 4).

**Figure 1:**
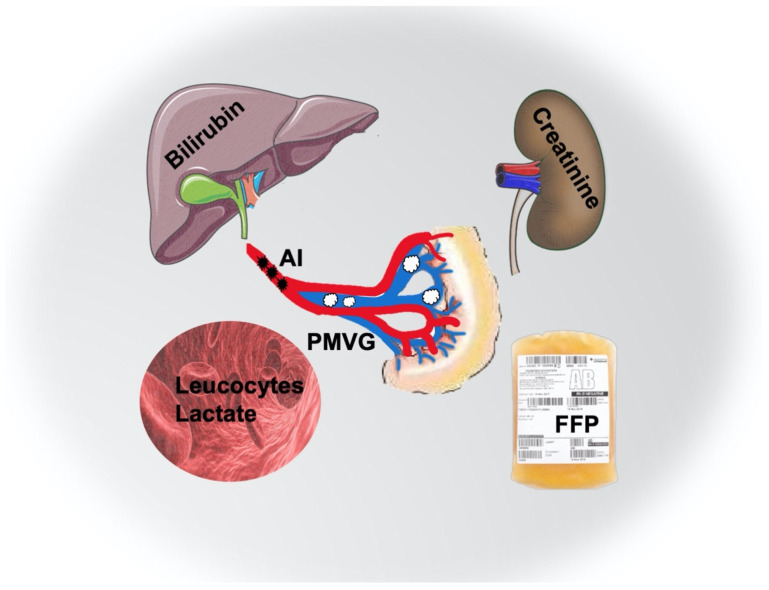
Major risk factors for mortality acute mesenteric ischemia. The graphical synopsis summarizes the major risk factors for mortality in acute mesenteric ischemia. Back dots indicate arterial occlusion, while white clouds indicate PMVG. AI, arterial ischemia; FFP, fresh frozen plasma; PMVG, portomesenteric vein gas.

All major risk factors for dismal outcome are also graphically presented in Figure 1.

## 4. Discussion

Despite a modern interdisciplinary treatment approach, the management and prevention of perioperative mortality are still challenging in AMI. Here, we aimed to evaluate the association of various clinico-pathological parameters with perioperative outcomes in patients with AMI undergoing surgical and/or interventional treatment. By conducting multivariate analyses, we identified leucocytes, bilirubin, creatinine and lactate, the presence of PMVG, AI and the intraoperative application of FFP as the most important predictors of outcome in our cohort.

In both multivariable models, the importance of lactate as a predictor of poor outcome was outlined. Although lactate levels have certain limitations for diagnosis purposes [7,8,18,19], an association between elevated lactate and inferior outcomes was described previously [11,20]. Here, we could demonstrate that postoperative mortality changes disproportionately with an increase in preoperative lactate levels (Table 4). Above 8 mmol/L at the time of initial diagnosis, a dismal in-house mortality of 95% was observed in our patients. However, our observation regarding lactate further underlines the prognostic and diagnostic dilemma in AMI patients. Almost one quarter of the cohort was diagnosed with AMI, despite lactate being within the physiological reference values and still displayed an in-house mortality of 25%. According to a meta-analysis from 2013, L-lactate, the isomer of lactate produced in anaerobic glycolysis, shows a pooled sensitivity of 86% and specificity of 44% in terms of diagnostic accuracy in patients with suspected AMI [18]. The latest guideline of the European Society of Vascular Surgery regarding the management of AMI rates L-lactate as too weak for diagnosing or ruling out an AMI [8]. In a further meta-analysis form 2017, D-lactate, produced due to bacterial fermentation, had a pooled sensitivity of 71.7%, but a specificity of 74.2% [19]. However, Nuzzo et al. showed, in a cross-sectional study from 2021, that D-lactate is not suitable for the differentiation of patients with AMI from patients with other acute abdominal pathologies [7]. In our cohort, lactate was measured preoperatively via venous blood gas analysis. Our findings underline that physiological serum lactate concentrations cannot be used to rule out neither an AMI nor a fatal outcome completely but if elevated, serum lactate provides a broadly available and feasible predictive marker.

Although this study did not aim to evaluate the diagnostic capabilities of radiological imaging for the diagnosis of AMI, we were still able to demonstrate a significant prognostic value of PVMG in our patients. The role of pathological signs within a CT scan for AMI has been examined previously. Emile et al. identified various radiological signs (PI, bowel distention, portomesenteric vein thrombosis and free intraperitoneal fluid) as predictors of existing bowel necrosis in AMI patients in a meta-analysis [21]. One larger multicenter study identified PMVG in combination with PI as a strong predictor for mortality independently from etiology [22]. In our univariate analysis, bowel distension and PI were also associated with mortality but did not achieve significance in the multivariable models. This might be explained by the notable morality in individuals presenting with PMVG (95.0%). However, the prognostic value is hampered by the relatively low prevalence (11.2%), indicating that PMVG is associated with a progressed AMI, subsequently resulting in fatal outcomes.

Despite the known role of lactate, also other laboratory parameters showed relevance in at least one of our multivariable models. The preoperative leukocyte count was an independent predictor for mortality in both multivariate analyses. Interestingly, the relevance of leukocyte count as prognostic parameter in AMI is not consistent throughout the literature. Although their prognostic value has been shown in some studies [12,13], other reports failed to show an association with mortality [23]. Furthermore, we were able to demonstrate serum creatinine and serum bilirubin as independent factors of postoperative mortality in our cohort. Renal impairment at initial diagnosis as prognostic factor was already shown in previous studies [16,24]; however, bilirubin has not been identified as a prognostic marker in AMI before. Of note, both parameters were not significant in the multivariable model excluding patients who deceased during primary surgery. This circumstance leads to the hypothesis, especially for bilirubin, that the elevation of these parameters is a sign of the onset of organ failure due to the septic constellation more likely than a direct malperfusion of the liver as a consequence of an accompanying occlusion of the TC. As part of the SOFA (sequential organ failure assessment) score, bilirubin and creatinine are commonly associated with higher mortality rates in septic patients independently from etiology [25].

Another prognostic factor in our analysis was the intraoperative administration of FFPs. During the study period, FFP was intraoperatively applied in cases of present coagulopathy. FFP transfusion is a known predictor of morbidity and mortality in gastrointestinal surgery [26]. One explanation might be the effect of transfusion-related immunomodulation. As such, Sarani et al. found a correlation between the transfusion of FFP and pulmonary or blood stream infections in critically ill surgical patients [27]. Some investigators speculated that soluble proteins in FFP may cause similar immunosuppressive effects, as seen in the case of red blood cell transfusions [27,28]. Potential mechanisms include diminished antigen processing by macrophages, the upregulation of both T suppressor/regulatory cells and humoral immunosuppressive mediators, impaired natural killer cell activity and the production of anti-idiotypic antibodies [28]. This is especially interesting as FFP was only significant in the secondary multivariable model after the exclusion of patients with fatal prognosis determined by the initial surgical exploration. Therefore, the above proposed effects of FFPs might in fact contribute to the inferior outcomes of initially treatable patients.

The importance of etiology subtypes as predictors for different outcomes was already identified by other groups [6,14]. As in our study, VI patients have a significantly better postoperative outcome compared to AI and NOMI. Interestingly, this better prognosis was observed, despite a higher median time to treatment in the VI group compared to other subtypes. It is, therefore, assumable that the time to irreversible bowel ischemia resulting in AI and NOMI patients is more rapid compared to the VI patients [29,30,31]. Interestingly, the time from diagnosis to treatment did not show prognostic value in our cohort at all, which is in contrast to previous reports underlining a short time to surgical treatment as a protective factor [4,5]. However, this might be explained by the small variety in time to treatment, limiting detectability within our used statical approach. Furthermore, AMI subtypes determine the extent of bowel resection in our cohort, which is line with the published literature [1]. In VI cases, the small bowel; in NOMI cases, mostly the colon; and in patients with AI, both the small intestine and colon in a similar distribution were evaluated as irreversibly damaged in the primary operation. Although no prognostic relevancy in multivariable analysis was observed in our patients, it must be considered that the extent of bowel resection is associated with long-term morbidity in surviving patients due to short bowel syndrome and high output enterostomies.

As with all retrospective clinical outcome studies, our analysis certainly has some obvious limitations, which have to be discussed. All data were collected in a retrospective fashion over a study period of more than ten years. Additionally, the patient treatment was carried out in accordance with our institutional clinical standards but not based on a defined study protocol, which carries an increased risk of selection bias and limits our conclusions. Due to the nature of AMI, a large set of patients deceased during the therapeutical process, with some patients considered palliative during initial exploration. To address the issue of these palliative patients within the dataset, we conducted two separate analyses including and excluding patients who did not undergo a curative treatment approach. Further, we are not able to elaborate on the time frame between the onset of symptoms and treatment as these data were not obtainable for a notable number of patients in this retrospective study. Additionally, our approach to include the full spectrum of AMI (AI, VI, and NOMI) combined with a limited dataset did not allow us to construct a valid preoperative risk score to predict outcome and guide treatment decisions.

Notwithstanding the limitations, we identified the degree of organ dysfunction (kidney and liver) and serum lactate, as well as radiological characteristics, of disease severity (PMVG), the underlying etiology (AI, NOMI) and intraoperative FFP administration to be of major importance for the prognosis of patients with AMI. As all these factors, except for FFP administration, are determined preoperatively and at the time of presentation, prognosis in these patients appears to be based on pretreatment characteristics. Furthermore, this underlines the importance of shortening the time to diagnosis of AMI. Unfortunately, the search for a valid biomarker has been and will be a challenge in upcoming years. In the above-mentioned cross-sectional study from 2021, D-lactate, intestinal fatty acid-binding protein and citrulline as three of the most promising biomarkers for early-stage AMI failed to distinguish patients with AMI from patients with acute abdominal pain of another origin [7]. The prevention and early diagnosis of AMI (e.g., through novel biomarkers and composite risk-assessment scores) seem to be of fundamental importance to improve outcomes in these patients and should be the focus of further research.

## Figures and Tables

**Table 1:** Patient characteristics with respect to disease etiology.

Variables	Overall Cohort (*n* = 179)	Arterial (*n* = 104)	Venous (*n* = 21)	NOMI (*n* = 54)
Demographics				
Gender, m/f, *n* (%)	87 (48.6)/92 (51.4)	46 (44.2)/58 (55.8)	10 (47.6)/11 (52.4)	31 (57.4)/23 (42.6)
Age, years	71 (60–81)	75 (63–82)	65 (49–69)	71 (59–78)
BMI, kg/m^2^	26 (23–29)	25 (22–28)	29 (27–37)	26 (24–29)
ASA, *n* (%)				
I	1 (0.6)	0	1 (4.8)	0
II	13 (7.3)	8 (7.7)	5 (23.8)	0
III	126 (70.4)	78 (75)	13 (61.9)	35 (64.8)
IV	37 (20.7)	17 (16.3)	2 (9.5)	18 (33.3)
Etiology, *n* (%)				
Embolic		57 (54.8)		
Thrombotic		41 (39.4)		
Compression		4 (3.8)		
Dissection		1 (1)		
Unknown		1 (1)		
Occluded vessel, *n* (%)				
TC		3 (2.9)		
SMA		66 (63.5)		
IMA		8 (7.7)		
TC+ SMA		19 (18.3)		
TC+ IMA		1 (1)		
SMA+ IMA		5 (4.8)		
TC+ SMA+ IMA		2 (1.9)		
Location of occlusion, *n* (%) *				
Proximal		72 (69.2)		
Distal		31 (29.8)		
Refferal from another hospital, *n* (%)	53 (29.6)	35 (33.7)	11 (52.4)	7 (13.2)
Radiological characteristics				
Pneumatosis intestinalis, *n* (%)	48 (26.8)	23 (22.1)	1 (4.8)	24 (44.4)
PMVG, *n* (%)	20 (11.2)	10 (9.6)	0	10 (18.5)
Bowel distension, *n* (%)	80 (44.7)	42 (40.4)	5 (23.8)	33 (61.1)
Bowel wall thickening, *n* (%)	99 (55.3)	49 (47.1)	18 (85.7)	32 (59.3)
Pneumoperitoneum, *n* (%)	21 (11.7)	8 (7.7)	0	13 (24.1)
Ascites, *n* (%)	56 (31.3)	16 (15.4)	15 (71.4)	25 (46.3)
Preoperative laboratory values				
Leukocytes, 1/nL	15.2 (10.9–23.5)	14.9 (10.6–22.9)	14.9 (10.9–26.9)	17.4 (10.4–23.8)
C-Reactive-Protein, mg/L	127 (37–230)	127 (25–230)	84 (53–161)	157 (97–197)
Hemoglobin, g/dL	12.0 (9.4–13.7)	12.1 (10.8–14.0)	13.9 (12.6–16.2)	9.0 (8.2–12.0)
Thrombocytes, 1/nL	228 (142–329)	249 (160–354)	294 (175–359)	149 (113–239)
Prothrombin time, %	70 (52–82)	72 (55–85)	71 (54–83)	64 (49–78)
INR	1.25 (1.12–1.53)	1.24 (1.10–1.49)	1.25 (1.10–1.47)	1.30 (1.17–1.59)
Bilirubin, mg/dL	0.7 (0.4–1.2)	0.7 (0.5–1.1)	0.9 (0.3–1.5)	0.8 (0.4–1.5)
AP, U/L	89 (69–129)	87 (67–112)	85 (72–120)	116 (70–174)
GGT, U/I	45 (24–93)	38 (23–87)	49 (23.3–114.3)	59 (34–132)
Albumin, g/dL	2.5 (1.8–3.4)	3.3 (1.9–3.8)	3.0 (2.5–3.6)	2.0 (1.6–2.7)
AST, U/L	44 (26–115)	40 (25–111)	28 (22–37)	88 (37–208)
ALT, U/L	38 (20–102)	31 (18–105)	25 (19–39)	51 (26–216)
Creatinine, mg/dL	1.3 (0.9–2.2)	1.3 (0.9–2.1)	1.1 (0.7 –1.5)	1.5 (1.0–3.2)
Lactate, mmol/L	3.3 (1.8–6.5)	3.3 (1.9–6.4)	2.3 (1.2–3.6)	4.0 (1.9–9.3)
Therapy Characteristics				
Extent of bowel resection, *n* (%)				
Small bowel	57 (31.8)	28 (26.9)	20 (95.2)	9 (16.7)
Colon	56 (31.3)	27 (26.0)	0	29 (53.7)
Small bowel and colon	30 (16.8)	20 (19.2)	0	10 (18.5)
No resection	12 (6.7)	11 (10.6)	1 (4.8)	0
Fatal	24 (13.4)	18 (17.3)	0	6 (11.1)
Technique of revascularization, *n* (%)				
Endovascular	27 (15.1)			
Open	40 (22.3)			
Thrombectomy	30 (17.8)			
Bypass, prosthetic,	4 (2.2)			
antegrade				
Bypass, prosthetic,	4 (2.2)			
retrograde				
Bypass, autologous vein,	2 (1.2)			
retrograde				
Combination	4 (2.2)			
Sequence of therapy				
Revascularization before resection	17 (9.5)			
Resection before revascularization	20 (11.2)			
Simultaneous	18 (10.1)			
Enterostomy, *n* (%)	124 (69.3)	68 (60.6)	15 (71.4)	46 (85.2)
Primary bowel anastomosis, *n* (%)	14 (7.8)	7 (6.7)	5 (23.8)	2 (3.7)
Intraoperative FFP transfusion, *n* (%)	34 (19)	12 (11.5)	3 (14.3)	19 (35.2)
Intraoperative blood transfusion, *n* (%)	69 (38.5)	38 (36.5)	6 (28.6)	25 (46.3)
Primary treatment time, minutes	130 (99–180)	129 (100–179)	124 (99–179)	140 (95–180)
Time to treatment, minutes	191 (110–363)	162 (100–269)	593 (315–770)	189 (113–339)
Intensive care stay, days	4 (1–15)	3.5 (1–14)	8 (2–28)	4 (1–16)
Postoperative data				
Postoperative complications, *n* (%)				
Clavien–Dindo I	0	0	0	0
Clavien–Dindo II	13 (7.3)	8 (7.7)	4 (19.1)	1 (1.9)
Clavien–Dindo IIIa	9 (5)	3 (2.9)	2 (9.5)	4 (7.4)
Clavien–Dindo IIIb	17 (9.5)	11 (10.6)	4 (19.1)	2 (3.7)
Clavien–Dindo IVa	19 (10.6)	10 (9.6)	1 (4.8)	8 (14.8)
Clavien–Dindo IVb	19 (10.6)	10 (9.6)	6 (28.6)	3 (5.6)
Clavien–Dindo V	100 (55.9)	61 (58.7)	3 (14.3)	36 (66.7)

Data presented as median and interquartile range, if not noted otherwise. * Vessel occlusions proximal from the first branch of the vessel were defined as “proximal”, while occlusions distal to the first branch were defined as “distal”. ALT, alanine aminotransferase; AP, alkaline phosphatase: ASA, American Society of Anesthesiologists classification; AST, aspartate aminotransferase; BMI, body mass index; CCI, comprehensive complication index; FFP, fresh frozen plasma; GGT, gamma glutamyltransferase; IMA; inferior mesenteric artery; INR, international normalized ratio; NOMI, non-occlusive mesenteric ischemia; PMVG, portomesenteric vein gas; SMA, superior mesenteric artery; TC, celiac trunk.

**Table 2:** Univariable and multivariable analysis of in-hospital mortality (overall cohort).

Variable			Univariable			Multivariable	
	*n*	Hazard Ratio	95% CI	*p*-Value	Hazard Ratio	95% CI	*p*-Value
Sex				0.905			
Age		1.02	1–1.05	**0.040**			0.961
BMI, kg/m^2^				0.440			
ASA				**0.004**			0.124
I/II	15	1					
III/IV	162	20.89	2.68–162.77				
Leukocytes, 1/nL		1.04	1.01–1.07	**0.025**	1.08	1.02–1.15	**0.008**
C-Reactive-Protein, mg/L				0.808			
Lactate, mmol/L		1.45	1.25–1.69	**<0.001**	1.25	1.05–1.47	**0.010**
Hemoglobin, g/dL		0.90	0.81–0.99	**0.048**			0.361
Albumin, g/L				0.832			
AST, U/L				0.077			
ALT, U/L				0.653			
GGT, U/L				0.178			
Bilirubin, mg/dL		1.6	1.06–2.41	**0.026**	2.05	1.02–4.12	**0.045**
Alkaline phosphatase, U/L		1.01	1–1.01	**0.034**			
Platelet count, 1/nL				0.290			
Prothrombin time, %		0.97	0.96–0.98	**<0.001**			0.377
INR		2.13	1.18–3.85	**0.012**			0.724
Etiology				**0.001**			**0.038**
Arterial	104	1			1		
Venous	21	0.12	0.03–0.42		0.12	0.02–0.89	
NOMI	54	1.41	0.71–2.8		0.97	0.32–2.97	
Pneumatosis intestinalis				**0.007**			0.774
No	121	1					
Yes	48	2.74	1.32–5.68				
Portomesenteric vein gas				**0.005**			**0.012**
No	149	1			1		
Yes	20	18.25	2.38–139.85		23.02	2.01–263.11	
Bowel Distension				**0.030**			0.838
<6 cm	89	1					
≥6 cm	80	1.99	1.07–3.69				
Bowel wall thickening				0.074			
Ascites				0.415			
Pneumoperitoneum				0.575			
Extent of resection				**0.003**			0.284
Small bowel	57	1					
Colon	56	1.48	0.7–3.13				
Small bowel and colon	30	4.38	1.66–11.53				
No resection in primary operation	12	1.14	0.32–4.03				
Fatal	24	>10	0–n.a.				
Treatment time, minutes				0.655			
Blood transfusions				0.190			
Intraoperative FFP transfusion				**0.004**			0.118
No	144	1					
Yes	34	3.75	1.54–9.16				
Time to treatment				0.128			
Referral from another hospital				0.841			

Various parameters are associated with postoperative mortality. All variables showing statistical significance in univariate binary logistic regression were included in a multivariable logistic regression. Hazard ratios are shown for statistically significant variables. AP was excluded in the multivariable analysis due to low case numbers. Bold indicates statistical significance. ALT, alanine aminotransferase; AP, alkaline phosphatase: ASA, American Society of Anesthesiologists classification; AST, aspartate aminotransferase; BMI, body mass index; FFP, fresh frozen plasma; GGT, gamma glutamyltransferase; INR, international normalized ratio; NOMI, non-occlusive mesenteric ischemia.

**Table 3:** Multivariable analysis of in-hospital mortality (fatal situation in primary operation excluded).

Variable		Mortality	
	Hazard Ratio	95% CI	*p*-Value
Age, years			0.961
ASA			0.159
Leucocytes, 1/nL	1.09	1.03–1.15	**0.004**
Lactate, mmol/L	1.27	1.08–1.48	**0.003**
Hemoglobin, g/dL			0.361
Bilirubin, mg/dL			0.166
Prothrombin time, %			0.377
INR			0.724
Creatinine, mg/dL			0.710
Etiology			**0.024**
AI	1		
VI	0.08	0.01–0.49	
NOMI	0.71	0.24–2.1	
Pneumatosis intestinalis			0.774
PMVG	17.02	1.62–178.58	**0.018**
Bowel distension			0.838
Extent of resection			0.233
Intraoperative FFP transfusion	4.4	1.2–16.11	**0.025**

All variables showing statistical significance in univariate binary logistic regression were included in a multivariable logistic regression. In this analysis, patients with a fatal result in the primary operation were excluded. Hazard ratios are shown for statistically significant variables. Bold values indicate statistical significance. AI, arterial ischemia; ASA, American Society of Anesthesiologists classification; INR, international normalized ratio; NOMI, non-occlusive mesenteric ischemia.; PMVG, portomesenteric vein gas; VI, venous ischemia.

**Table 4:** Univariable analysis of in-hospital mortality divided in lactate subgroups.

Variable			Mortality	
	*n*	Hazard Ratio	95% CI	*p*-Value
Lactate, mmol/L				
≤2	48	1		<0.001
>2; ≤4	57	2.52	1.09–5.80	0.030
>4; ≤8	34	9.75	3.49–27.23	<0.001
>8	32	45	9.33–217.04	<0.001

Statistical increasing risk of in-hospital mortality with increasing preoperative lactate values demonstrated in 4 subgroups.

## Data Availability

The data presented in this study are available upon request from the corresponding author. The data are not publicly available due to privacy reasons.

## Supplementary Materials

The following supporting information can be downloaded at: https://www.mdpi.com/article/10.3390/jcm11133619/s1. Table S1. Sub-analysis for patients with arterial ischemia with respect to revascularization.
