# Supplementary material for: Prognostic Factors for Mortality in Acute Mesenteric Ischemia
Source: J Clin Med. 2022 Jun 23;11(13):3619. doi: 10.3390/jcm11133619 (PMC9267588; doi:10.3390/jcm11133619)
Supplement: Supplementary file 1 [file jcm-11-03619-s001.zip › jcm-1735976-supplementary.pdf]

Table S1. Sub-analysis for patients with arterial ischemia with respect to revascularization.

Variables	Arterial ischemia (revascularized, n=63)	Arterial ischemia (not-revascularized, n=27)	p value
**Demographics**			
Gender, m/f, n (%)	24 (38.1)/39 (61.9)	19 (70.4)/8 (29.6)	.005
Age, years	72 (62-79)	81 (63-84)	.047
BMI, kg/m^2	25.7 (22.6-29)	24.2 (22.26.7)	.351
ASA, n (%)			.625
I	0	0	
II	6 (9.5)	2 (7.4)	
III	50 (79.4)	20 (74.1)	
IV	7 (11.1)	5 (18.5)	
Etiology, n (%)			.002
Embolic	39 (61.9)	9 (29.6)	
Thrombotic	24 (38.1)	13 (48.1)	
Compression	0	4 (14.8)	
Dissection	0	0	
Unknown	0	1 (3.7)	
Occluded vessel, n (%)			.000
TC	1 (1.6)	1 (3.7)	
SMA	45 (71.4)	14 (51.9)	
IMA	0	8 (29.6)	
TC+ SMA	14 (22.2)	2 (7.4)	
TC+ IMA	2 (3.2)	0	
SMA+ IMA	0	2 (7.4)	
TC+ SMA+ IMA	1 (1.6)	0	
Location of occlusion, n (%)*			.034
Proximal	47 (74.6)	14 (51.8)	
Distal	16 (25.4)	13 (48.1)	
Refferal from another hospital, n (%)	27 (42.9)	4 (14.8)	.010
**Radiological characteristics**			
Pneumatosis intestinalis, n (%)	6 (9.5)	7 (25.9)	.080
PMVG, n (%)	2 (3.2)	3 (11.1)	.184
Bowel distension, n (%)	23 (36.5)	9 (33.3)	.459
Bowel wall thickening, n (%)	29 (46)	13 (48.1)	.697
Pneumoperitoneum, n (%)	3 (4.8)	3 (11.1)	.355
Ascites, n (%)	8 (12.7)	5 (18.5)	.643
**Preoperative laboratory values**			
Leukocytes, 1/nl	16 (11.4-23.4)	11.9 (8.7-18.2)	.090
C-Reactive-Protein, mg/l	137.5 (24.4-230)	141 (36.7-258.5)	.804
Hemoglobin, g/dl	12.1 (10.9-14)	12.1 (10.5-14.1)	.836
Thrombocytes, 1/nl	259 (167-354)	242 (159-282)	.315
Prothrombin time, %	74.5 (51-96.50)	71.5 (56.8-78.3)	.310
INR	1.17 (1.02-1.56)	1.25 (1.14-1.45)	.194
Bilirubin, mg/dl	.74 (.5-1.14)	.59 (.36-.97)	.174
AP, U/l	87 (67.5-104.5)	86 (65-108)	.908
GGT, U/l	37 (24-65.5)	46 (22-122)	.577
Albumin, g/dl	3 (1.9-3.6)	3.5 (3.1-5.2)	.130
AST, U/l	40 (26-111)	31 (20-45.5)	.041
ALT, U/l	38 (25-316)	17 (14-28)	.002
Creatinine, mg/dl	1.04 (0.8-1.5)	2.3 (1.57-3.4)	.000
Lactate, mmol/l	2.9 (1.6-5.9)	3.3 (1.9-5.1)	.610
**Therapy Characteristics**			
Extent of bowel-resection, n (%)			.001
Small Bowel	24 (38.1)	4 (14.8)	

Colon	10 (15.9)	17 (63)	
Small Bowel and Colon	14 (22.2)	6 (22.2)	
No Resection	11 (17.5)	0	
Fatal	4 (6.3)	0	
Technique of revascularization, n (%)			
Endovascular	23 (36.5)		
Open	36 (57.1)		
Thrombectomy	26 (41.3)		
Bypass, prosthetic, antegrade	4 (6.3)		
Bypass, prosthetic, retrograde	4 (6.3)		
Bypass, autologous vein, retrograde	2 (3.17)		
Combination	4 (6,3)		
Sequence of therapy			
Revascularization before resection	17 (27)		
Resection before revascularization	13 (20.6)		
Simultaneous	18 (28.6)		
Enterostomy, n (%)	42 (66.6)	26 (96.3)	.008
Primary bowel anastomosis, n (%)	6 (9.5)	1 (3.7)	.345
Intraoperative FFP transfusion, n (%)	8 (12.7)	3 (11.1)	.813
Intraoperative blood transfusion, n (%)	23 (36.5)	13 (48.1)	.329
Primary treatment time, minutes	152 (120-205)	127 (115-173)	.132
Time to treatment, minutes	160 (95-261)	215 (152-346)	.027
Intensive care stay, days	6 (2-15)	4	.849
**Postoperative data**			
Postoperative complications, n (%)			
Clavien-Dindo >IIIa	59 (93.7)	22 (81.5)	.343
Inhospital mortality	33 (52.4)	14 (51.9)	.963

Patients deemed palliative during initial exploration were excluded from the analysis. Data presented as median and interquartile range, if not noted otherwise. *Vessel occlusions proximal from the first branch of the vessel were defined as "proximal", while occlusions distal to the first branch were defined as "distal". ALT, alanine aminotransferase; AP, alkaline phosphatase: ASA, American society of anesthesiologists classification; AST, aspartate aminotransferase; BMI, body mass index; CCI, comprehensive complication index; FFP, fresh frozen plasma; GGT, gamma glutamyltransferase; IMA; inferior mesenteric artery; INR, international normalized ratio; PMVG, portomesenteric vein gas; SMA, superior mesenteric artery; TC, celiac trunk
